# The role of α-E-catenin in cerebral cortex development: radial glia specific effect on neuronal migration

**DOI:** 10.3389/fncel.2014.00215

**Published:** 2014-08-07

**Authors:** Marie-Theres Schmid, Franziska Weinandy, Michaela Wilsch-Bräuninger, Wieland B. Huttner, Silvia Cappello, Magdalena Götz

**Affiliations:** ^1^Helmholtz Zentrum München, National Research Center for Environmental Health, Institute of Stem Cell ResearchNeuherberg/Munich, Germany; ^2^Max Planck Institute of Molecular Cell Biology and GeneticsDresden, Germany; ^3^Department of Physiological Genomics, Institute of Physiology, University of MunichMunich, Germany

**Keywords:** α-E-catenin, actin, neuronal migration, cell polarity, subcortical band heterotopia, adherens junctions

## Abstract

During brain development, radial glial cells possess an apico-basal polarity and are coupled by adherens junctions (AJs) to an F-actin belt. To elucidate the role of the actin, we conditionally deleted the key component α-E-catenin in the developing cerebral cortex. Deletion at early stages resulted in severe disruption of tissue polarity due to uncoupling of AJs with the intracellular actin fibers leading to the formation of subcortical band heterotopia. Interestingly, this phenotype closely resembled the phenotype obtained by conditional RhoA deletion, both in regard to the macroscopic subcortical band heterotopia and the subcellular increase in G-actin/F-actin ratio. These data therefore together corroborate the role of the actin cytoskeleton and its anchoring to the AJs for neuronal migration disorders.

## Introduction

Malformations of the human neocortex represent a major cause of developmental disabilities and severe epilepsy (Barkovich et al., 2012). It is therefore essential to understand how these diseases develop, find the critical molecules, discover gene networks, and pathways relevant for these malformations and deeply comprehend the cellular functions and the functional consequences.

A feature of the mammalian neocortex is the positioning of functionally distinct neurons in six horizontal layers (Leone et al., 2008). The importance of this arrangement becomes particularly evident in cases of disturbed neocortical development, like in subcortical band heterotopias (SBH), in which neurons remain located below the white matter. This and other malformations are the result of impaired developmental processes such as cell proliferation, differentiation or neuronal migration (Bozzi et al., 2012).

Recent evidence shows that the formation of neuronal heterotopias can also result from defect in the polarity and morphology of radial glial cells (Cappello et al., 2012, 2013; Cappello, 2013), highlighting the emerging role of radial glia polarity and architecture in the etiology of cortical malformations and in particular neuronal migration disorders.

Apical radial glial cells in the developing nervous system possess epithelial features such as junctional coupling at the ventricular surface, localize distinct molecules to the apical and basolateral membrane domain and contact the basement membrane by their radially extending processes (Gotz and Huttner, 2005). On the contrary, the intermediate or basal progenitors, differ from apical radial glial cells as they lack apico-basal polarity and mostly divide once to generate two postmitotic neurons (Haubensak et al., 2004; Miyata et al., 2004; Noctor et al., 2004; Wu et al., 2005). Intrinsic molecules regulating apico-basal polarity in radial glial cells may be involved in orchestrating the complex process of neurogenesis and neuronal migration. Indeed, alterations in cell polarity and adherens junction (AJ) coupling upon conditional deletion of cdc42 from radial glial cells lead to an immediate transition of radial glial cells to basal progenitors (Cappello et al., 2006). However, it is not clear to which extent the phenotype is due to the influence of cdc42 on the Par complex (Costa et al., 2008), on AJ coupling or the random cell localization.

Recently genetic deletion of RhoA in the developing cerebral cortex highlighted the essential role of radial glial cells and AJs localization as it resulted in the formation of subcortical band heterotopia and Lissencephaly type II, with neurons protruding beyond layer I at the pial surface of the brain (Cappello et al., 2012).

We therefore aimed here to selectively examine the significance of junctional coupling on apical radial glial cells in the developing cerebral cortex. Toward this end, we conditionally deleted the AJ molecule, α-E-catenin which has been shown to dynamically connect the F-actin cytoskeleton to β-catenin (Harris and Tepass, 2010), which in turn is bound to cadherins (Harris and Tepass, 2010). The loss of α-E-catenin was reported to cause AJ disassembly (Vasioukhin et al., 2001; Lien et al., 2006) and widespread deletion of α-E-catenin in the CNS led to profound overproliferation and disturbances in tissue architecture (Lien et al., 2006). Conversely, deletion of α-N-catenin, mostly expressed in differentiated neurons, led to relatively mild defects in the CNS architecture (Park et al., 2002; Uemura and Takeichi, 2006). However, in addition to its role in junctional coupling, α-E-catenin was reported to affect signaling pathways, such as MAP kinase in skin stem cells (Vasioukhin et al., 2001), WNT in HEK293T cells (Merdek et al., 2004) and Sonic Hedgehog (SHH) signaling in radial glial cells (Lien et al., 2006). The deletion of α-E-catenin in the entire nervous system affected amongst others the signaling centers for SHH, the ventral most regions throughout the CNS (Jessell and Sanes, 2000). We choose to delete α-E-catenin specifically in the developing cerebral cortex, avoiding the ventral SHH signaling center, by using the Emx1^Cre^ mouse line (Iwasato et al., 2000; Cappello et al., 2006, 2012). These experiments support a key role of α-E-catenin in coupling AJ complexes to the apical radial glia cells. When this link is abolished, tissue polarity is lost and newly generated neurons fail to reach the cortical plate and accumulate in ectopic positions resulting in the formation of a large subcortical band heterotopia.

Therefore this result strengthens the hypothesis that subcortical band heterotopia is a disorder caused by tissue defects in radial glial cells rather than in the migration of neurons themselves (Cappello et al., 2012; Cappello, 2013).

## Materials and methods

### Animals

The mouse strains Catna1^tm1Efu^ (Vasioukhin et al., 2001) was obtained from Jackson Laboratory. The Emx1^Cre^ mouse strain was kindly provided by Iwasato et al. (2000). All mouse strains were maintained on a C57BL/6JxCD1 background. Analysis was performed from E10 to P0. Embryonic brains were dissected in HBSS/10 mM HEPES (Gibco-Invitrogen), fixed in 4% paraformaldehyde (PFA), cryoprotected with 30% sucrose and frozen sections were cut at 12 μm thickness for immunohistochemistry or *In Situ*-hybridization.

### BrdU-labeling

Pregnant mice were injected intraperitoneally at E11, E12, and E14 with 5-Bromo-2-deoxyuridine (BrdU) (5 mg/kg bodyweight) and analyzed by immunostaining at P0, (birth dating analysis) as previously shown (Cappello et al., 2012). Quantification was not performed.

### F- to G-actin analysis and quantification

F- and G-actin were labeled by Texas-Red labeled Phalloidin (0.165 μM, Invitrogen) and Alexa 488-labeled DNase1 (9 μg/ml, Mol.Probes) respectively. Cryosections (fixed in 4%PFA) were incubated with both reagents for 20 min, washed in PBS and additionally immunolabeled for Tuj1. Images were taken by confocal microscopy and quantitatively analyzed using ImageJ (Java).

### Immunohistochemistry and *in situ*-hybridization

Primary antibodies: α-catenin (1:20, BD-Transd.Lab), pan-cadherin (1:200, Sigma), Par3 (1:100, Upstate), Prominin (1:100, eBioscience), Tuj1 (1:100, Sigma), Tbr2 (1:100, Chemicon/Millipore), Calretinin (1:5000, SWant), BrdU (1:100, Abcam), reelin (1:10, Merck), Tbr1 (1:100, Abcam), β-catenin (1:50, BD-Trans.D.), RC2 (provided by P.Leprince), NeuN (1:100, Chemicon/Millipore), L1 (1:2000, Chemicon/Millipore), Nurr1 (1:200, Santa Cruz). Secondary antibodies were used form Jackson Immunoresearch, and Southern Biotechnology. Nuclei were visualized by using DAPI (4′, 6′Diamidino-2-phenylindoline, 0.1 μg/ml,Sigma) or PropidiumIodide (Mol. Probes). Specimens were mounted in Aqua Poly/Mount (Polysciences, Northampton, UK) and analyzed with FV1000 (Olympus) or Axioplan/ApoTome Microscope (Zeiss).

Digoxigenin-labeled RNA probes for ER81, Cux2 (kindly provided by C.Schuurmans), Satb2, (kindly provided by V. Tarabykin), and Rorβ (Armentano et al., 2007) were made and used as described (Chapouton et al., 2001).

### Electron microscopy

Electron microscopy was performed as described previously (Cappello et al., 2006).

### Western Blot

Primary antibodies: Par3 (1:500, Chemicon/Millipore), aPKCλ, i (1:500, Cell Signaling), Par6 (1:500, Santa Cruz), Phospho-aPKCλ (1:500 Cell Signaling).

Cortices from embryonic brains were lysed in RIPA buffer. 20 μg of total protein were separated by 10% SDS-PAGE and transferred to PVDF membranes (Biorad), which were incubated with primary antibodies followed by horseradish peroxidase labeled secondary antibodies (1:25000; Amersham) detected by ECL Western Blotting Detection (Chemicon).

## Results

**Figure 1:**
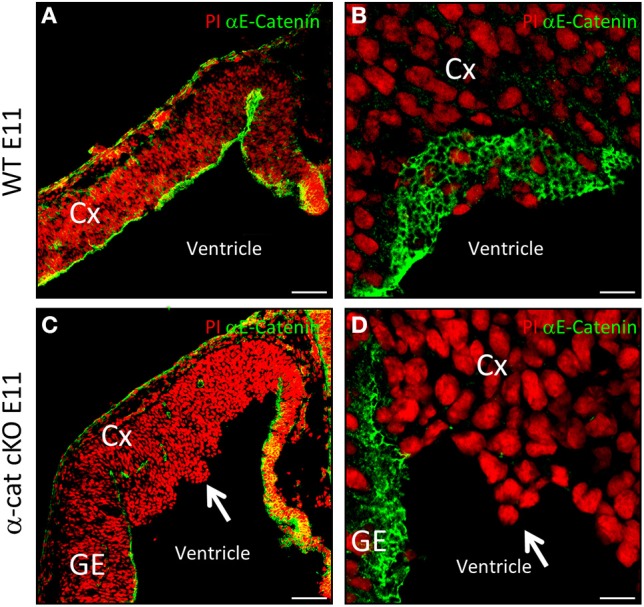
**Cortical development upon loss of α-catenin**. α-E-catenin immunostaining (green, nuclei labeled with Propidium Iodide, red) in coronal sections of E11 WT **(A,B)** and α-cat cKO **(C,D)** cortices. Note the loss of α-E-catenin upon recombination by Emx1^Cre^ from E11 on, exclusively in the cortex, except in blood-vessels, the meninges, and the Choroid Plexus. Arrows indicates protrusions in the lateral ventricle. CX, cortex; GE, ganglionic eminence. Scale bars: 100 μm **(A,C)**, 10 μm **(B,D)**.

In radial glial cells α-E-catenin localizes to AJs (Figure 1A), visible as honeycomb-like structures when viewed from the ventricular surface (Figure 1B). We investigated the function of α-E-catenin by deleting exon3 of α-E-catenin (Vasioukhin et al., 2001) in radial glial cells selectively in the cerebral cortex at early stages of neurogenesis by Emx1^Cre^ (Iwasato et al., 2000; Cappello et al., 2006, 2012). Accordingly, α-E-catenin was lost from the anlagen of the cerebral cortex in Emx1^Cre/α−catΔex2fl/fl^ mutant mice (referred to as α-cat cKO henceforth) but was unaffected in the remaining CNS, including the ganglionic eminence that contains the SHH signaling center (Figures 1C,D). Consistent with the onset of Cre-expression in Emx1^Cre^ cortices around E9.5, α-E-catenin mRNA started to decrease at E10 and α-cat cKO cortices lacked α-E-catenin protein at E11 (Figures 1C,D). Already at E11 the mutant cortex showed morphological alterations, with cells starting to protrude into the ventricle (Figures 1C,D, arrows).

### Neurons migrate in two bands in α-cat cKO cortex and form a double-cortex

**Figure 2:**
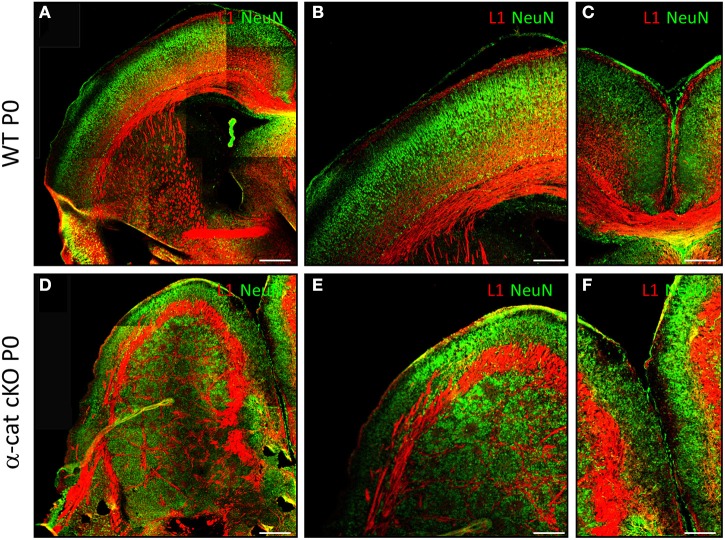
**Formation of SBH in the absence of α-E-catenin**. Fluorescence micrographs of coronal cortical sections of P0 WT **(A–C)** and α-cat cKO **(D–F)** immunostained as indicated. **(A,B,D,E)**: lateral cortex; **(B,E)**: enlarged view of **(A,D)**; **(C,F)**: connection between the two hemispheres [corpus callosum in **(C)**]. Scale bars: 200 μm **(A,D)**, 100 μm **(B,C,E,F)**.

We next examined α-cat cKO cortices at postnatal stages, when most of the neurons have reached the correct final position. At postnatal day 0 (P0) in WT brains, neurons, positive for the neuronal marker NeuN, are located in the cortical plate and separated from the ventricular zone by the white matter containing L1 positive axons (Figures 2A,B). α-cat cKO cortices also displayed a similar band of L1 positive axons that separated a thin layer of NeuN positive neurons correctly positioned in the cortical plate but also had a large heterotopia below composed of neurons organized in clusters which were surrounded by L1 positive axons (Figures 2D,E). This double band of gray matter neurons (split by white matter) and the additional white matter intermingled in the heterotopia demonstrated that deletion of α-E-catenin in the developing cortex resulted in the formation of a large SBH or double-cortex. Moreover, L1 positive axons did not form a corpus callosum connecting the two cerebral hemispheres (Figures 2C,F).

### Both parts of the double-cortex possess neurons from all layers

**Figure 3:**
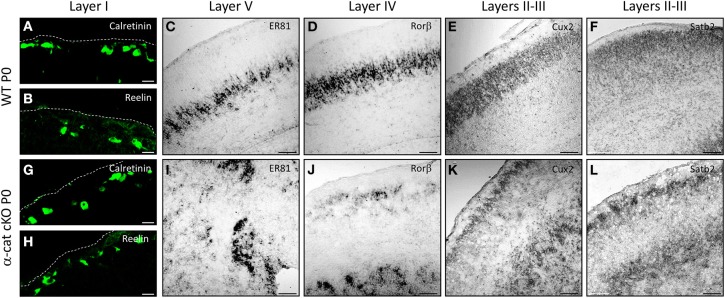
**Characterization of the SBH cell identity in the α-cat cKO cortex**. Micrographs of coronal sections of P0 WT **(A–F)** and α-cat cKO **(G–L)**. Micrographs depict immunostaining (Calretinin, Reelin) or *in situ* hybridization (ER81, rorβ, cux2, satb2) as depicted in the panels. **(A,B,G,H)**: pial surface is indicated by dashed lines. Scale bars: 10 μm **(A,B,G,F)**; 100 μm **(C–F,I–L)**.

In order to determine the identity of the neurons located at the different (normal and aberrant) positions, neuronal subtype specific markers were examined after birth (P0). The earliest generated neurons (E10/11) populate layer I and express Calretinin and/or Reelin (Stoykova et al., 2003; Englund et al., 2005). Both in WT and α-cat cKO cortices these neurons were situated at correct positions in layer I beneath the pial surface (Figures 3A,B,G,H). However subplate neurons, expressing Nurr1, were distributed both in the upper cortex and the heterotopia (Figure S1), suggesting that the neuronal heterotopia is formed very early during development. Neurons generated a little later (E12/13), normally settle in layers IV and V and express Rorβ and ER81 (Figures 3C,D). These neurons appeared sometimes in clusters in the α-cat cKO cortex (Figures 3I,J) and despite being generated early, already showed a distinct distribution in an upper band and clusters located below the white matter. More clearly, neurons generated later, that settle in the upper cortical layers II/III and express Cux2 and Satb2 (including partially layer IV) (Nieto et al., 2004; Zimmer et al., 2004; Britanova et al., 2005) (Figures 3E,F), were present in two well defined bands located in the upper cortex and clustered in the heterotopia in the P0 α-cat cKO cortex (Figures 3K,L). These data suggest that both, the upper and the lower, cortices were composed by neurons of each layer including the subplate.

**Figure 4:**
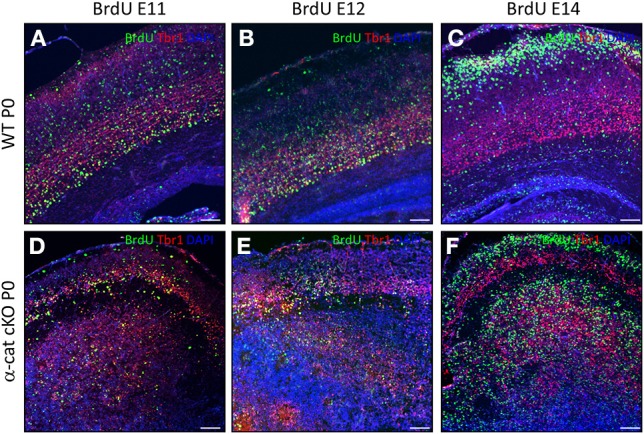
**Birth-dating analysis in the α-cat cKO cortex**. Fluorescence micrographs of coronal cortical sections of P0 WT **(A–C)** and α-cat cKO **(D–F)** immunostained as indicated. BrdU was injected at E11 **(A,D)**, E12 **(B,E)**, or E14 **(C,F)**. Scale bars: 100 μm.

In WT, neurons of different layers are generated sequentially at different developmental stages. As some of the above used markers may also be altered in the mutant, we examined whether in the α-cat cKO cortex neurons were still generated in the same order, BrdU was administered to pregnant mice at day E11, E12, or 14 and brains were analyzed at P0. In WT, a large proportion of BrdU-positive cells generated at E11 expressed the deep layer neuronal marker Tbr1 and was located in the deep layers (Figure 4A). Neurons generated at E11 in the α-cat cKO were mostly positive for Tbr1 but spread in the entire cortex up to layer I and the pial surface, including a prominent band in the cortical plate (Figure 4D). Neurons generated at E12, mostly expressed the deep layer marker Tbr1 in both WT and α-cat cKO cortices (Figures 4B,E), whereas neurons generated at E14 in WT were mostly negative for Tbr1 (Figure 4C). This colocalization with Tbr1 was similar in the α-cat cKO cortex (Figure 4F). Thus, neurons generated from α-E-catenin deficient progenitor cells acquired their neuronal identity according to their time of generation and independently of their position within the cortex. This suggests that the fate of radial glial cells and basal progenitors does not depend on their residence in particular cortical niches.

In contrast to WT however, the vast majority of neurons born at E12 (Tbr1+) or at E14 (Tbr1−) were located in two separated locations in the α-cat cKO cortex (Figures 4E,F).

These data together demonstrate that deletion of α-E-catenin in the developing cerebral cortex resulted in the formation of SBH.

### Neural stem cell position and morphology are altered in α-cat cKO cortex

**Figure 5:**
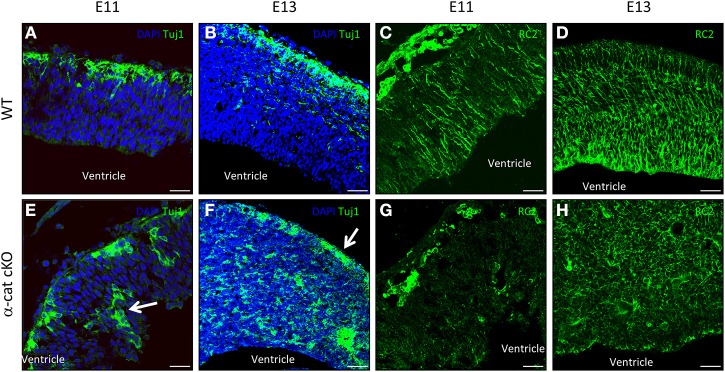
**Neuronal position and stem cell morphology upon loss of α-catenin**. Fluorescence micrographs of coronal cortical sections of E11 and E13 WT **(A–D)** and α-cat cKO **(E–H)** immunostained as indicated. Arrows indicate ectopic neurons **(E)** or neurons reaching the cortical plate **(F)**. Scale bars: 50 μm **(A,C,E,G)**, 100 μm **(B,D,F,H)**.

Previous work suggested that maintenance of radial glial cell polarity is essential to guide neurons toward the cortical plate (Cappello et al., 2012), we therefore analyzed radial glial cells and the localization of newly-generated neurons (Tuj1+) at earlier stages in the α-cat cKO developing cortex. As neurogenesis in the mouse cerebral cortex begins around E10, the position of neurons was investigated 1 day later when α-E-catenin protein was already strongly reduced in the α-cat cKO cortex. At this stage in WT only a thin band of neurons positive for Tuj1 is populating the cortical plate as most of the cells are still proliferating (Figure 5A). Later on, at E13, the cortical plate becomes thicker and the newly-generated neurons together with their future axons occupy almost half of the entire cortex (Figure 5B). Interestingly already at E11, neurons were localized at ectopic positions in the α-cat cKO, including the ventricular zone (Figure 5E, arrow), suggesting that neuronal migration toward the cortical plate was impaired. Two days later, only few Tuj1 positive neurons managed to reach the cortical plate (Figure 5F, arrow) while most of them accumulated in ectopic positions scattered in the developing cortex (Figure 5F).

As the current hypothesis is that aberrant radial glia polarity is the cause of defective neuronal migration (Cappello et al., 2012; Cappello, 2013), immunostaining for the RC2 isoform of the intermediate filament nestin (Chanas-Sacre et al., 2000) was performed. Immunoreactivity for RC2 was almost absent in the α-cat cKO cortex at E11 (Figures 5C,G), and later on, at E13, highlighted a complete disruption of the radial glial morphology in the α-cat cKO suggesting that the loss of α-E-catenin results in an impaired cytoskeletal organization in radial glial cells (Figures 5D,H) as in the case of the RhoA cKO (Cappello et al., 2012).

### Cortical cells maintain cell-cell contacts in the absence of α-E-catenin

**Figure 6:**
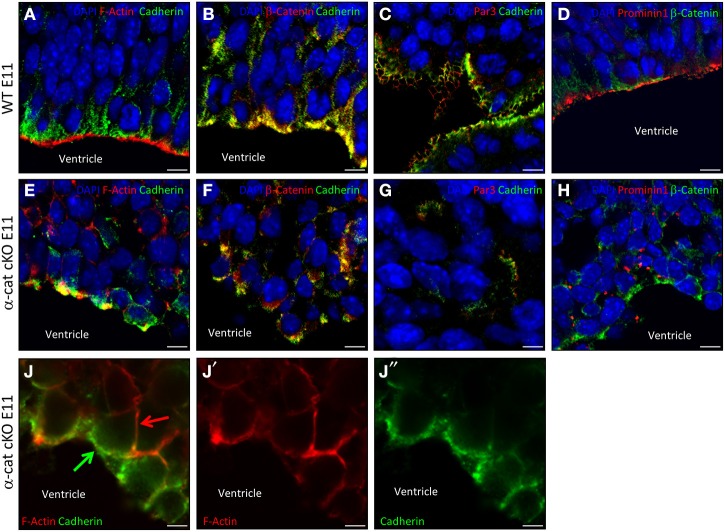
**Adhesion and polarity upon loss of α-catenin**. Fluorescence micrographs of coronal cortical sections of E11 WT **(A–D)** and α-cat cKO **(E–J)** immunostained as indicated. Arrows indicate uncoupled AJs (green) and F-actin (red) in α-cat cKO cortices **(J)**. Scale bars: 10 μm **(A–H)**, 5 μm **(J–J″)**. Student's *t*-test, ^*^P < 0.05.

The cellular alterations, exemplified by the scattering and intermingling of cells, were a prominent aspect of the α-cat cKO phenotype and strongly suggested that the loss of α-E-catenin affected cellular coherence. To better understand the cell biological consequences of the α-E-catenin deletion, cell adhesion, junctional coupling, and the cytoskeleton were then examined in the α-cat cKO cortex. In WT cortices cadherin-immunoreactivity delineated the AJs around progenitors along the ventricle (Figures 6A–C) where it co-localized with β-catenin, adjacent to the concentrated rings of F-actin (Figure 6A). While disorganized patches of cadherin-staining were irregularly distributed within the tissue of α-cat cKO cortices (Figures 6E,J), they still co-localized with β-catenin (Figure 6F), suggesting that AJs may still be present between cortical cells, despite the loss of α-E-catenin even though in a random manner. This impression was confirmed at the ultra-structural level where long electron dense structures were present between α-E-catenin-deficient cells (Figure S2). However, these structures were not regularly aligned at the ventricular surface as in WT cortices (Figure S2B), but rather detected throughout the cortex parenchyma. The cell-cell junctions present in the α-cat cKO cortex were found either dispersed solitarily or in clusters at different sites (Figures 6F,G). The largest clusters formed rosette-like structures (data not shown) similar to what has been observed in other mutants (Lien et al., 2006; Kadowaki et al., 2007; Rasin et al., 2007; Cappello et al., 2012).

The dispersal of cells and the absence of a radial cortical architecture suggested disturbances of the cytoskeletal organization in the absence of α-E-catenin. Indeed, despite the presence of AJs, the coupling between AJs and intracellular actin fibers was strongly compromised as already at E11 the accumulation of F-actin was not concentrated at the same side as cadherin (Figures 6E,J). This is consistent with the role of α-E-catenin as an anchor to connect the transmembrane AJs to the cytoskeleton by linking the β-catenin/cadherin complex to the intracellular actin fibers (Harris and Tepass, 2010). Thus, while nascent AJs are made, these fail to be anchored to the cytoskeleton.

Several polarity molecules are co-localized together with AJs. We therefore examined whether the alteration in the cytoskeletal organization may have abolished cell polarity. To address this issue we investigated molecules of the apical membrane domain, such as prominin1 or the members of the apical Par complex (Suzuki and Ohno, 2006; Srinivasan et al., 2008). Interestingly, WT and α-cat cKO cortices contained comparable levels of Par3, Par6, and aPKC, as well as phosphorylated aPKC (Figures 6C,G and Figure S3). Furthermore, Par3, although distributed in patches within the α-cat cKO cortex parenchyma, still localized next to cadherins (Figures 6C,G), suggesting that at least some degree of cellular polarity was maintained in the α-cat cKO cortex. This was further supported as Prominin1 (CD133), delineating the ventricular surface in WT (Weigmann et al., 1997), was concentrated in distinct patches along the cell membrane of mutant cells (Figures 6D,H), adjacent to β-catenin patches. Moreover, prominin-containing particles released from the apical membrane (Marzesco et al., 2005) were observed close to AJs within the α-cat cKO cortex (data not shown). Thus, α-E-catenin deficient cortical cells possess AJs and concentrate apical membrane components in their proximity, demonstrating that individual cells were able to, at least partially, maintain their polarity despite the intermingling of cell types and tissue architecture in the α-cat cKO.

### Deletion of α-E-catenin impairs the stability of F-actin at the ventricular surface

**Figure 7:**
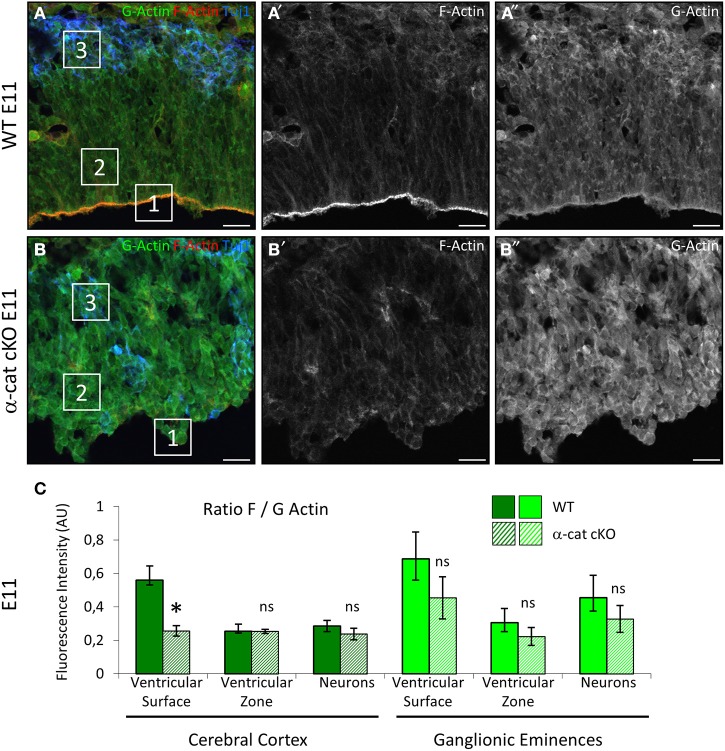
**Alterations in in the actin cytoskeleton**. Visualization of F- and G-actin in E11 WT and α-cat cKO cortices **(A,B)**. The ratios of F to G-actin were quantified by confocal microscopy **(C)**. Squares in **(A)** and **(B)** exemplify the regions in which the intensity of F- and G actin was measured. Student's *t*-test, ^*^P < 0.05. Scale bars: 20 μm.

α-E-catenin directly interacts with the cytoskeleton by binding to F-actin at the place of AJs and thereby stabilizing its filamentous form (Pokutta et al., 2008). Therefore it was investigated whether the absence of α-E-catenin may have changed the stability of filamentous actin, thus shifting the ratio between G- and F-actin toward G-actin, as this was the case upon conditional RhoA deletion (Cappello et al., 2012). The ratio of F- to G-actin was assessed by detecting F-actin with fluorescently labeled Phalloidin and G-actin with DNAseI. The fluorescent intensity was then quantified by confocal microscopy. This revealed an increase in G-actin in the α-cat cKO cortical VZ to levels found in the WT only in the cortical plate (Figures 7A–B″). The intensities of G- and F-actin were measured for progenitors at the ventricular surface (Figure 7A, square1) and within the parenchyma (Figure 7A, square2) and for neurons (Figure 7A, square3) separately. This revealed a specifically high ratio of F- to G-actin at the ventricular surface of WT cortices, where AJs are positioned. The area of progenitors within the tissue and neurons showed lower ratios of F to G-actin. The pronounced high ratio of F- to G-actin at the ventricular surface was much reduced in the α-cat cKO cortex and F- to G-actin ratios were rather homogeneous throughout the mutant cortex (Figures 7B,C). In contrast, F- to G-actin ratios remained unaltered in the GE where α-E-catenin expression is maintained (Figure 7C). These results may indicate a generally higher instability of actin filaments in the absence of α-E-catenin. The anchorage of the actin filaments to the AJs by α-E-catenin therefore may be critically required for the maintenance of a radial morphology in cortical progenitor cells.

## Discussion

### α-E-catenin cKO results in formation of double-cortex

This analysis of α-E-catenin function in the developing cerebral cortex revealed a surprising result, the formation of a prominent double-cortex or SBH that was not reported in previous work deleting α-E-catenin in the entire developing nervous system (Lien et al., 2006, 2008; Stocker and Chenn, 2006, 2009). Interestingly, neurons of all types except layer I were sequentially generated in the α-E-catenin cKO cortex and distributed in both the upper cortex and the lower heterotopia, suggesting that some neurons, even late generated, and destined for the upper layers, can reach their position in the normal cortex and migrate on top of the previously generated deep layers. Conversely other neurons generated at the same time settle in a rather more clustered/nuclear manner in the ectopic lower cortex. Interestingly, this phenotype closely resembles the conditional deletion of RhoA (Cappello et al., 2012) and thus suggests common mechanisms at the base of double-cortex formation.

In addition this phenotype also resembles the subcortical band heterotopia described in other mutants, like the HeCo mutant (Croquelois et al., 2009; Kielar et al., 2014) and the conditional mutant RA-GEF-1, a guanine nucleotide exchange factor (GEF) for Rap1 (Bilasy et al., 2009). While the latter mutant has not been yet analyzed in detail, the cellular mechanism of the double-cortex formation appears to be rather comparable in the HeCo, RhoA, and α-E-catenin mutants.

### Radial glial cell defects at the base of double-cortex formation

Both RhoA and α-E-catenin mutants have common morphological defects of radial glial cells. These occur at the apical side due to uncoupling of the AJs and the actin cytoskeleton. Already at E11, 1 day after recombination by Emx1^Cre^, the architecture of the cerebral cortex is disrupted in the α–cat cKO and the morphology of the radial glial cells is strongly affected. Later on, at E13, it is already possible to notice that RC2+ processes are not anymore spanning from the apical to the basal side but start forming round-shape structures, namely rosettes (see Figure 5H). These rosettes-like structures persist later in development, at P0 (see Figure 2D) and accumulate largely in the heterotopic cortex (see Figure 2E). But why do rosettes form?

From the earliest stages of either α-E-catenin or RhoA deletion, the ventricular lining, normally kept together by junctional coupling, starts to get disorganized. In both mutants cadherins can still bind to each other and form nascent AJs as observed by ultrastructural analysis. However these can no longer sustain larger pressure exerted by growth and bulging out of the dorsal telencephalon. In both, the conditional RhoA and α-E-catenin mutants this displaces clusters of radial glial cells that are still coupled to areas of less pressure namely inside the cerebral cortex thereby forming rosettes. Within these rosettes radial glial cells are still coupled by adherens junctional belts and have an apical membrane domain oriented toward the lumen of the rosette. These cells generate neurons migrating to the outer region of the rosette where they then often settle. This is the basis of the formation of the lower double-cortex with neurons arranged in clusters. Notably, the white matter of these rosettes does not localize, as is the case in the normal cortex, between the cell bodies of radial glial cells and the neurons, but around the rosettes, confining the physical space of a rosette. This may be due to the shorter length of the radial glial cells and radial extension of the rosettes and/or the strength by which the radial processes could resist a fiber tract forming between their basal and apical anchoring (see also below). Indeed, where and how the basal processes of the radial glial cells in the rosettes anchor is still ill understood. One possibility is the basement membrane at blood vessels in the vicinity but this remains to be shown.

Some rosettes, however, also get to be localized at more superficial positions with radial glial cells still extending some shorter processes to the surface (see also Cappello et al., 2012). This allows some neurons even generated at late stages still to reach the upper, normotopic part of the cortex. Importantly, these neurons can then migrate through pre-existing layers settling on top, as both in the RhoA, α-E-catenin cKO, and HeCo mutants neurons in the normotopic cortex are organized in an inside first outside later layering.

Interestingly, the comparison between the RhoA (Cappello et al., 2012) and a-cat cKO shows that the timing of rosette formation seemingly determines the size of the double-cortex. In the α-E-catenin mutant the rupture of the apical surface and rosette formation occurs earlier (already around E11/12), while this reorganization takes longer in the RhoA cKO (Cappello et al., 2012). As a consequence more neurons manage to follow intact radial glial processes in the RhoA cKO and reach the correct position in the cortical plate and therefore a thicker normotopic cortex is formed compared to the α-E-catenin mutant (Cappello et al., 2012). According to the earlier defects the α-E-catenin cortex displays a very large heterotopia and a thin layered cortex which is probably why it was overlooked in earlier studies. Accordingly, in the α-E-catenin double-cortex model, also the early-generated subplate neurons are clearly localized in both the upper cortex and the large heterotopia. On the contrary both the HeCo and RhoA mutants have a larger upper layered cortex and smaller heterotopia compared to the α-E-catenin mutant (Croquelois et al., 2009; Cappello et al., 2012).

### Effects on the actin cytoskeleton affect the maintenance of adherens junctions strength and radial processes

The earlier onset of the phenotype in the α-cat cKO compared to the RhoA cKO may be due to the slightly longer perdurance of the RhoA protein (no longer detectable by immunostaining at E12) compared to α-E-catenin (no longer detectable by immunostaining at E11). As the same Cre-line was used for genetic recombination this may be due to protein stability or differences in the efficiency of recombining the different loci. In addition, the functional consequences for the strength of AJ coupling may be more severe when α-E-catenin is not present as the key anchoring molecule between the cadherins at the cell surface and the F-actin belts is lost. RhoA clearly affects the stabilization of actin filaments (Govek et al., 2011; Cappello et al., 2012) but even after complete loss of the protein this may result in less severe defects than the loss of α-E-catenin.

Interestingly, the formation and/or stabilization of actin filaments is also deficient upon loss of α-E-catenin. This may be a consequence of the uncoupling between AJs and actin filaments. As less F-actin is engaged in anchoring to the AJs to the cytoskeleton, actin filaments are disassembled and produce more globular actin. However, actin instability is also the cause for destabilization of the radial processes where no AJs are present, suggesting that the loss of α-E-catenin has effects on the actin filaments throughout the cell. It is also possible that α-E-catenin regulates the dynamics of actin by modulating the activity of Arp2/3 (Drees et al., 2005) or by binding formin-1, an actin nucleator (Kobielak et al., 2004). Indeed, this is the same in the RhoA cKO, where radial glia processes are also destabilized due to changes in the F- to G-actin ratio (Cappello et al., 2012). Upon loss of RhoA also the stabilization of microtubules is affected, suggesting another possible mechanism mediated by microtubules. This is consistent with the requirement of fine-tuning the microtubules stability for migration and process formation in neurons (Creppe et al., 2009; Heng et al., 2010). Moreover, in order to preserve the proper morphology, radial glial cells may require a general stabilization of the cytoskeleton that can be maintained either by actin fibers or stable microtubules (Li et al., 2003; Vreugdenhil et al., 2007) or the intermediate filaments. Intriguingly in both RhoA and α-E-catenin mutants, as well as in the HeCo mutant, Nestin (RC2) was severely downregulated.

Neurons may also change the direction of migration upon cytoskeleton destabilization as for instance Lamellipodin depletion caused pyramidal neurons to adopt a tangential, rather than radial glial guided migration mode by reducing the activity of Serum Response Factor and therefore the ratio of polymerized to unpolymerized actin (Pinheiro et al., 2011).

All together these data suggest that a general destabilization of the cytoskeleton, either actin or microtubules or both, in radial glial cells and neurons can influence their key role in maintaining tissue architecture and their radial processes. Either change can be fatal for neurons that use the glial cells as a guidance to find the cortical plate.

### Conflict of interest statement

The authors declare that the research was conducted in the absence of any commercial or financial relationships that could be construed as a potential conflict of interest.

## Supplementary material

Figure S1 **Characterization of the SBH cell identity in the α-cat cKO cortex**. Micrographs of coronal sections of P0 WT **(A,A′)** and α-cat cKO **(B,B′)**. Micrographs depict Nurr1 immuno- and DAPI- staining as indicated in the panels. Scale bars: 100 μm.

Figure S2 **Adherens Junctions upon loss of α-catenin**. Electron micrographs of WT **(A)** and α-cat cKO **(B,B′)** E11 cortices revealing electrondense AJs (indicated by arrows). Ventricle is down in **(A)**. **(B′)**: high magnification of boxed area in **(B)**. Scale bars: 500 nm.

Figure S3 **Par complex upon loss of α-catenin**. Western Blot of cortical tissue from E11 WT and α-cat cKO. Primary antibodies used for the comparison are indicated above the lanes, lower strips blotted for β-actin serve as loading controls.
