# Supplementary figures and images for: The role of α-E-catenin in cerebral cortex development: radial glia specific effect on neuronal migration
Source: Front Cell Neurosci. 2014 Aug 7;8:215. doi: 10.3389/fncel.2014.00215 (PMC4124588; doi:10.3389/fncel.2014.00215)

Figure S1

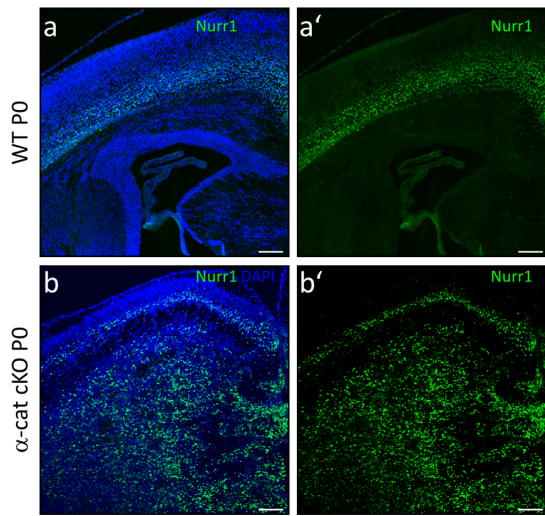

Supplement: Figure S1 — Characterization of the SBH cell identity in the α-cat cKO cortex. Micrographs of coronal sections of P0 WT (A,A′) and α-cat cKO (B,B′). Micrographs depict Nurr1 immuno- and DAPI- staining as indicated in the panels. Scale bars: 100 μm. [file Presentation1.PDF]

Figure S2

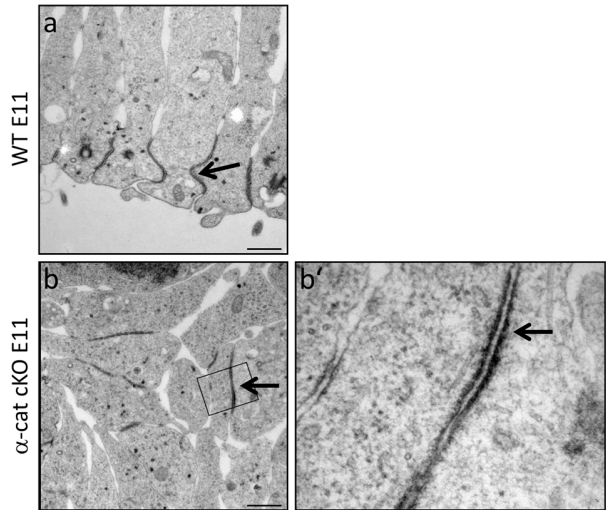

Supplement: Figure S2 — Adherens Junctions upon loss of α-catenin. Electron micrographs of WT (A) and α-cat cKO (B,B′) E11 cortices revealing electrondense AJs (indicated by arrows). Ventricle is down in (A). (B′): high magnification of boxed area in (B). Scale bars: 500 nm. [file Presentation2.PDF]

Figure S3

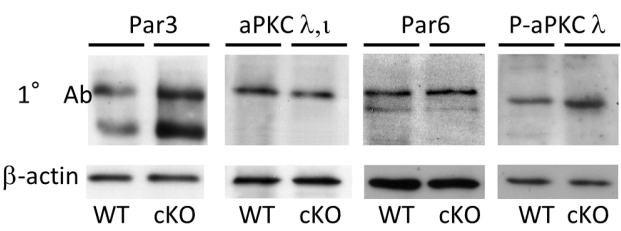

Supplement: Figure S3 — Par complex upon loss of α-catenin. Western Blot of cortical tissue from E11 WT and α-cat cKO. Primary antibodies used for the comparison are indicated above the lanes, lower strips blotted for β-actin serve as loading controls. [file Presentation3.PDF]
